# Supplementary material for: ProteinShader: illustrative rendering of macromolecules
Source: BMC Struct Biol. 2009 Mar 30;9:19. doi: 10.1186/1472-6807-9-19 (PMC2672931; doi:10.1186/1472-6807-9-19)
Supplement: Additional file 1 — ProteinShader program without source code. This compressed file contains the complete ProteinShader program including associated libraries, but no source code. A README.txt file gives an overview of the ProteinShader distribution, and the index.html file in the help subdirectory has directions on getting started with the program as well as a set of tutorials. [file 1472-6807-9-19-S1.zip › ProteinShader-beta-0_9_4-binary/help/api/org/proteinshader/structure/io/exceptions/StructureReaderException.html]

StructureReaderException (ProteinShader API)


|  |  |  |
| --- | --- | --- |
| **Overview** \| **Package** \| **Class** \| **Use** \| **Tree** \| **Deprecated** \| **Index** \| **Help** | |  |
| **PREV CLASS**   NEXT CLASS | **FRAMES**    **NO FRAMES**     **All Classes** |  |
| SUMMARY: NESTED \| FIELD \| CONSTR \| METHOD | DETAIL: FIELD \| CONSTR \| METHOD |  |


---


## org.proteinshader.structure.io.exceptions Class StructureReaderException

```
java.lang.Object
  java.lang.Throwable
      java.lang.Exception
          org.proteinshader.structure.io.exceptions.StructureReaderException
```

**All Implemented Interfaces:**: Serializable

---

``` public class StructureReaderException extends Exception ```

Used to report that an error occurred while reading a file to create a
Structure.

**See Also:**: Serialized Form

---

**Constructor Summary**

`StructureReaderException()`
  
          Sets the default message to "An error occurred while reading the
file".

`StructureReaderException(String message)`
  
          The message given this constructor can be retrieved using
getMessage().


**Method Summary**

**Methods inherited from class java.lang.Throwable**

`fillInStackTrace, getCause, getLocalizedMessage, getMessage, getStackTrace, initCause, printStackTrace, printStackTrace, printStackTrace, setStackTrace, toString`

**Methods inherited from class java.lang.Object**

`clone, equals, finalize, getClass, hashCode, notify, notifyAll, wait, wait, wait`

**Constructor Detail**

### StructureReaderException

```
public StructureReaderException()
```

:   Sets the default message to "An error occurred while reading the
    file".
    This message can be retrieved using getMessage().

---


### StructureReaderException

```
public StructureReaderException(String message)
```

:   The message given this constructor can be retrieved using
    getMessage().

    **Parameters:**: `message` - a description of the problem.


---


|  |  |  |
| --- | --- | --- |
| **Overview** \| **Package** \| **Class** \| **Use** \| **Tree** \| **Deprecated** \| **Index** \| **Help** | |  |
| **PREV CLASS**   NEXT CLASS | **FRAMES**    **NO FRAMES**     **All Classes** |  |
| SUMMARY: NESTED \| FIELD \| CONSTR \| METHOD | DETAIL: FIELD \| CONSTR \| METHOD |  |


---

# *Copyright © 2007-2008*
